# Association of advanced glycation end products with sarcopenia and frailty in chronic kidney disease

**DOI:** 10.1038/s41598-020-74673-x

**Published:** 2020-10-19

**Authors:** Junko Yabuuchi, Seiji Ueda, Sho-ichi Yamagishi, Nao Nohara, Hajime Nagasawa, Keiichi Wakabayashi, Takanori Matsui, Higashimoto Yuichiro, Tomoyasu Kadoguchi, Tomoyuki Otsuka, Tomohito Gohda, Yusuke Suzuki

**Affiliations:** 1 Department of Nephrology, Juntendo University Faculty of Medicine, 2-1-1 Hongo, Bunkyo-ku, Tokyo, 113-8421 Japan; 2 Division of Diabetes, Metabolism and Endocrinology, Department of Medicine, Showa University School of Medicine, Tokyo, Japan; 3 Department of Pathophysiology and Therapeutics of Diabetic Vascular Complications, Kurume University School of Medicine, Kurume, Japan; 4 Department of Chemistry, Kurume University School of Medicine, Kurume, Japan; 5 Department of Cardiovascular Medicine, Juntendo University Graduate School of Medicine, Tokyo, Japan

**Keywords:** Health care, Nephrology

## Abstract

Prevalence of sarcopenia is high in patients with chronic kidney disease (CKD), especially in those with dialysis. Various pathological conditions related to CKD, such as chronic inflammation, insulin resistance, and endothelial dysfunction, are thought to be associated with the development and progression of sarcopenia. Advanced glycation end products (AGE), one of the representative uremic toxins, have been shown to contribute to various CKD-associated complications. This study investigated the role of AGE in frailty and sarcopenia in patients and animals with CKD, respectively. In patients undergoing dialysis, serum AGE levels were significantly increased according to the frailty status and inversely associated with physical performance and activity. AGE accumulated in the gastrocnemius muscle of 5/6 nephrectomy mice in association with morphological abnormalities, capillary rarefaction, and mitochondrial dysfunction, all of which were completely inhibited by DNA-aptamer raised against AGE. Our present findings may suggest the pathological role of AGE in sarcopenia and frailty in CKD.

## Introduction

Sarcopenia is frequently observed in patients with chronic kidney disease (CKD), especially in those with end-stage renal failure on dialysis^1,2^. Indeed, the more severe renal dysfunction, the higher prevalence rate for sarcopenia^3^. In addition, sarcopenia is the most common cause of physical frailty in CKD patients, which is associated with various comorbidities and increased risk of mortality in these patients^4^. Frailty is primarily defined as cumulative physiological declines in functional reserve and resistance to stressors across the multiorgan systems that occur during the aging process^5^. CKD was originally known as one of the representative conditions that could accelerate premature aging^6^, and therefore various pathological conditions associated with CKD, such as chronic inflammation, insulin resistance, and increased uremic toxins, have been shown to contribute to the risk of aging-related disorders, including sarcopenia and frailty^3,7,8^.

Nonenzymatic modifications of amino groups of proteins or lipids by monosaccharides, such as glucose, glyceraldehyde, and fructose lead to formation and accumulation of advanced glycation end products (AGE) in the human body^9^. In addition to diabetic patients^10^, circulating AGE levels are increased in patients with renal dysfunction, which is partly ascribed to the impaired renal clearance of AGE in these patients^11^. Therefore, AGE are generally thought to represent a uremic toxin^12–14^. There is a growing body of evidence to show that AGE play a crucial role in CKD-associated complications, such as atherosclerotic cardiovascular disease, left ventricular hypertrophy, heart failure, and anemia^15–17^. The interaction of AGE with their receptor, receptor for AGE (RAGE) has been shown to evoke oxidative stress and inflammation, and is thereby involved in insulin resistance and endothelial dysfunction in patients with CKD^14,18,19^. Furthermore, increased levels of serum AGE are associated with poor grip strength and slow walking speed in older adults^20,21^. In diabetic patients, it has also been reported an inverse association between skin autofluorescence, a marker of tissue accumulation of AGE, with grip strength and knee extension strength^22^. Besides, higher serum levels of AGE are associated with prevalent frailty in older adults^23^. In addition, an in vitro-study revealed that AGE could induce muscle atrophy and impair myogenesis via a RAGE-mediated signaling pathway^24^. These observations led us to hypothesize that accumulation of AGE in CKD, one of the representative diseases that accelerate aging-related disorders, could contribute to the pathogenesis of sarcopenia and frailty in CKD as well. However, the detailed pathophysiological mechanisms regarding the relationship frailty and accumulated AGE in CKD or diabetes remain to be elucidated. In this study, we investigated the pathological role of AGE in frailty and sarcopenia in CKD patients and an animal model of CKD, respectively.

## Results

### Patients’ characteristics

**Figure 1:**
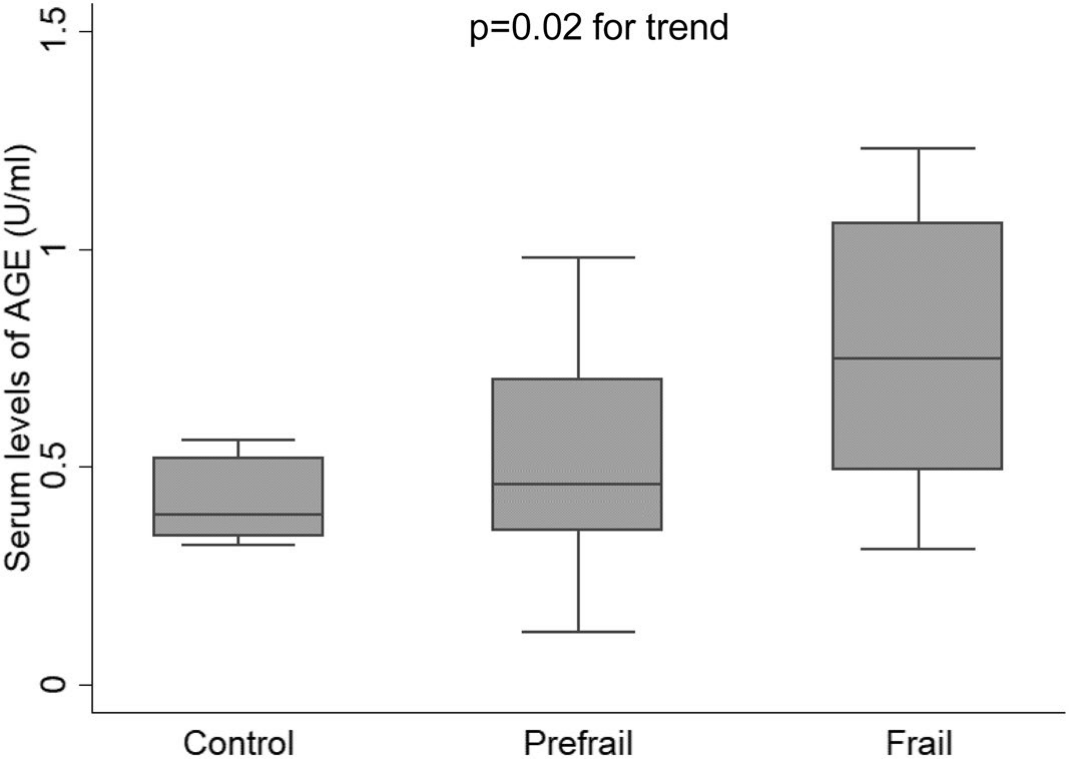
Box plot chart of serum level of AGE according to the frailty status. AGE values were significantly increased according to the degree of frailty (p = 0.02 for trend by ANOVA). The top and bottom of the boxes indicate 75 and 25 percentiles, respectively. The line through the middle of each box represents the median. The upper and lower error bars show the minimum and maximum values, respectively.

Table 1 shows the clinical characteristics of HD patients. Mean age of our patients (22 men and 15 women) was 67.2 years, and mean duration of dialysis was 8.9 years. Consistent with those in previous studies^25^, patients with diabetes (0.92 ± 1.25, n = 10) had increased serum AGE levels compared with non-diabetic subjects (0.56 ± 0.27, n = 25), with no statistically significant differences (p = 0.17). Of the patients, 22% met the definition of frail and 65% prefrail. Frailty was associated with older age (p < 0.01) and had a trend toward lower diastolic blood pressure and lower GNRI (p = 0.06 and p = 0.07, respectively). There were no statistically significant differences in other clinical characteristics and serum biochemistry among the three groups. When AGE levels were stratified by the frailty status, serum levels of AGE were significantly increased according to the degree of frailty (p = 0.02, by ANOVA) (Fig. 1). Mean AGE levels were significantly higher in patients with slowness or weight loss than those without (see Supplementary Table S1 online).

**Table 1:** Clinical characteristics of patients.

	Control	Prefrail	Frail	p-value
Patients (number)	5	24	8	
Male, % (number)	20 (1)	62 (15)	75 (6)	0.13
Age (years old)	64 ± 11	64 ± 9.5	79 ± 9.7	< 0.01
Duration of dialysis (years)	9.3 ± 9.0	9.2 ± 7.7	7.4 ± 5.4	0.61
**Primary disease, % (number)**				
CGN	60 (3)	29 (7)	25 (2)	0.36
Diabetic nephropathy	0	29 (7)	38 (3)	0.31
PCKD	0	13 (3)	13 (1)	0.70
Nephrosclerosis	20 (1)	25 (6)	25 (2)	0.97
Others	20 (1)	4.2 (1)	5.2 (0)	0.30
SBP (mmHg)	163 ± 13	166 ± 24	167 ± 30	0.81
DBP (mmHg)	86 ± 7.6	86 ± 11	75 ± 15	0.06
Diabetes, % (number)	0	38 (9)	38 (3)	0.25
Glycoalbumin (%)	14.9 ± 0.8	16.1 ± 3.8	16.4 ± 3.7	0.52
GNRI	94 ± 7.2	92 ± 5.8	87 ± 9.3	0.07
Triglycerides (mg/dL)	125 ± 71	143 ± 75	123 ± 41	0.89
HDL-C (mg/dL)	47 ± 5.1	48 ± 17	47 ± 10	0.99
LDL-C (mg/dL)	104 ± 21	91 ± 17	82 ± 29	0.10
Glucose (mg/dL)	95 ± 18	140 ± 55	139 ± 73	0.23
Albumin (g/dL)	3.7 ± 0.3	3.6 ± 0.3	3.4 ± 0.5	0.14
hsCRP (mg/dL)	0.4 ± 0.7	0.5 ± 1.0	0.3 ± 0.3	0.76
BUN (mg/dL)	63 ± 15	67 ± 16	72 ± 20	0.36
Creatinine (mg/dL)	9.9 ± 2.8	12 ± 2.2	9.5 ± 2.7	0.49
Corrected Ca (mg/dL)	9.0 ± 0.5	9.4 ± 0.6	9.3 ± 0.9	0.49
P (mg/dL)	4.9 ± 1.2	5.7 ± 1.1	6.1 ± 1.8	0.12
Intact-PTH (pg/mL)	113 ± 82	193 ± 150	205 ± 78	0.27

Values for continuous variables given as mean ± standard deviation.

*CGN* chronic glomerulonephritis, *PCKD* polycystic kidney disease, *SBP* systolic blood pressure, *DBP* diastolic blood pressure, *GNRI* Geriatric Nutritional Risk Index, *HDL-C* high-density lipoprotein cholesterol, *LDL-C* low-density lipoprotein cholesterol, *hsCRP* high-sensitivity C-reactive protein, *BUN* blood urea nitrogen, *Ca* calcium, *P* phosphorous, *PTH* parathyroid hormone.

### Correlation of AGE levels with physical performance and physical activity

As shown in Table 2, higher AGE levels were significantly associated with a longer time needed to complete the timed up and go (TUG) test (p = 0.03) and decreased average metabolic equivalents (METs) (p = 0.02).

**Table 2:** Correlation of AGE levels with physical performance and activity.

Physical performance		Correlation coefficient
**Grip strength**		
Right		r = − 0.10, n.s.
Left		r = − 0.19, n.s.
Timed up and go test		r = 0.41, p = 0.03
**One-leg standing duration**		
Right		r = − 0.18, n.s.
Left		r = − 0.27, n.s.
**Physical activity**		
Average metabolic equivalents		r = − 0.41, p = 0.02
Average steps		r = − 0.20, n.s.

Correlation coefficient was calculated by Spearman’s correlation test.

*n.s.* not significant.

### Effects of AGE-aptamer on clinical variables and CSA of gastrocnemius muscles in 5/6Nx CKD model mice

We further examined the role of AGE in sarcopenia in 5/6Nx mice, an animal model of CKD. The clinical characteristics of animals are shown in Table 3. BUN and creatinine levels were significantly elevated in 5/6Nx mice as compared with sham mice, whereas body weight was lower in CKD animals. Control-aptamer or AGE-aptamer treatment did not affect the renal function or body weight in 5/6Nx mice (Table 3).

**Table 3:** Clinical characteristics of mice.

	Sham	5/6Nx	5/6Nx-control-aptamer	5/6Nx-AGE-aptamer
Number	11	12	9	14
Blood urea nitrogen (mg/dl)	34.0 ± 8.4	67.2 ± 5.8*	68.4 ± 4.9*	66.0 ± 11.6*
Creatinine (mg/dl)	0.55 ± 0.15	1.29 ± 0.15*	1.02 ± 0.28*	1.43 ± 0.44*
Body weight (g)	28.4 ± 1.5	26.1 ± 1.2*	25.3 ± 0.8*	26.6 ± 1.6*
Gastrocnemius weight (g)	0.17 ± 0.01	0.16 ± 0.01	0.16 ± 0.01	0.16 ± 0.01

Data are shown as mean ± standard deviation. 5/6 Nx, 5/6 nephrectomy.

*p < 0.05 compared with sham mice.

**Figure 2:**
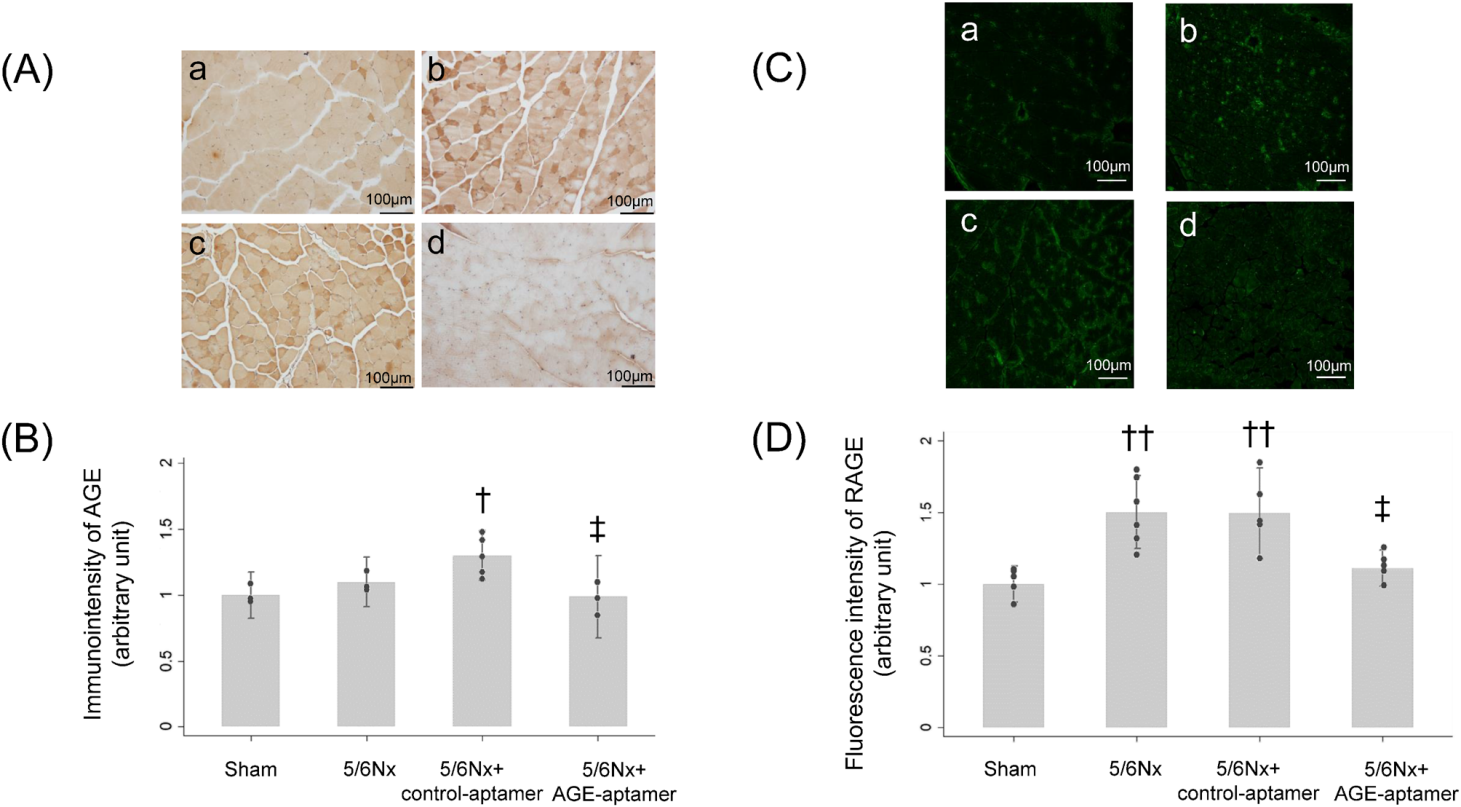
Immunohistochemical analysis for AGE and RAGE in the gastrocnemius muscles. (**A**) Representative photographs of AGE immunostaining in the gastrocnemius muscles. × 20 magnification. a, Sham mice; b, 5/6Nx mice; c, 5/6Nx mice treated with control-aptamer; d, 5/6Nx mice treated with AGE-aptamer. (**B**) Quantitative data of AGE staining. n = 3–5 per each group. ^†^p < 0.05 compared with sham mice. ^‡^p < 0.05 compared with 5/6Nx mice plus control-aptamer. (**C**) Representative microphotographs of RAGE immunostaining in the gastrocnemius muscles. × 20 magnification. a, Sham mice; b, 5/6Nx mice; c, 5/6Nx mice treated with control-aptamer; d, 5/6Nx mice treated with AGE-aptamer. (**D**) Quantitative data of RAGE staining. n = 5–6 per each group. ^††^p < 0.01 compared with sham mice. ^‡^p < 0.05 compared with 5/6Nx mice plus control-aptamer.

As shown in Fig. 2A,B, AGE levels in the gastrocnemius muscles of 5/6Nx mice treated with control-aptamer mice were significantly higher than those of sham mice, which was reduced by the treatment with AGE-aptamer. Furthermore, compared with sham mice, the RAGE levels in the gastrocnemius muscles of 5/6Nx and control-aptamer-treated 5/6Nx mice were significantly increased, but were also inhibited by AGE-aptamer treatment (Fig. 2C,D).

**Figure 3:**
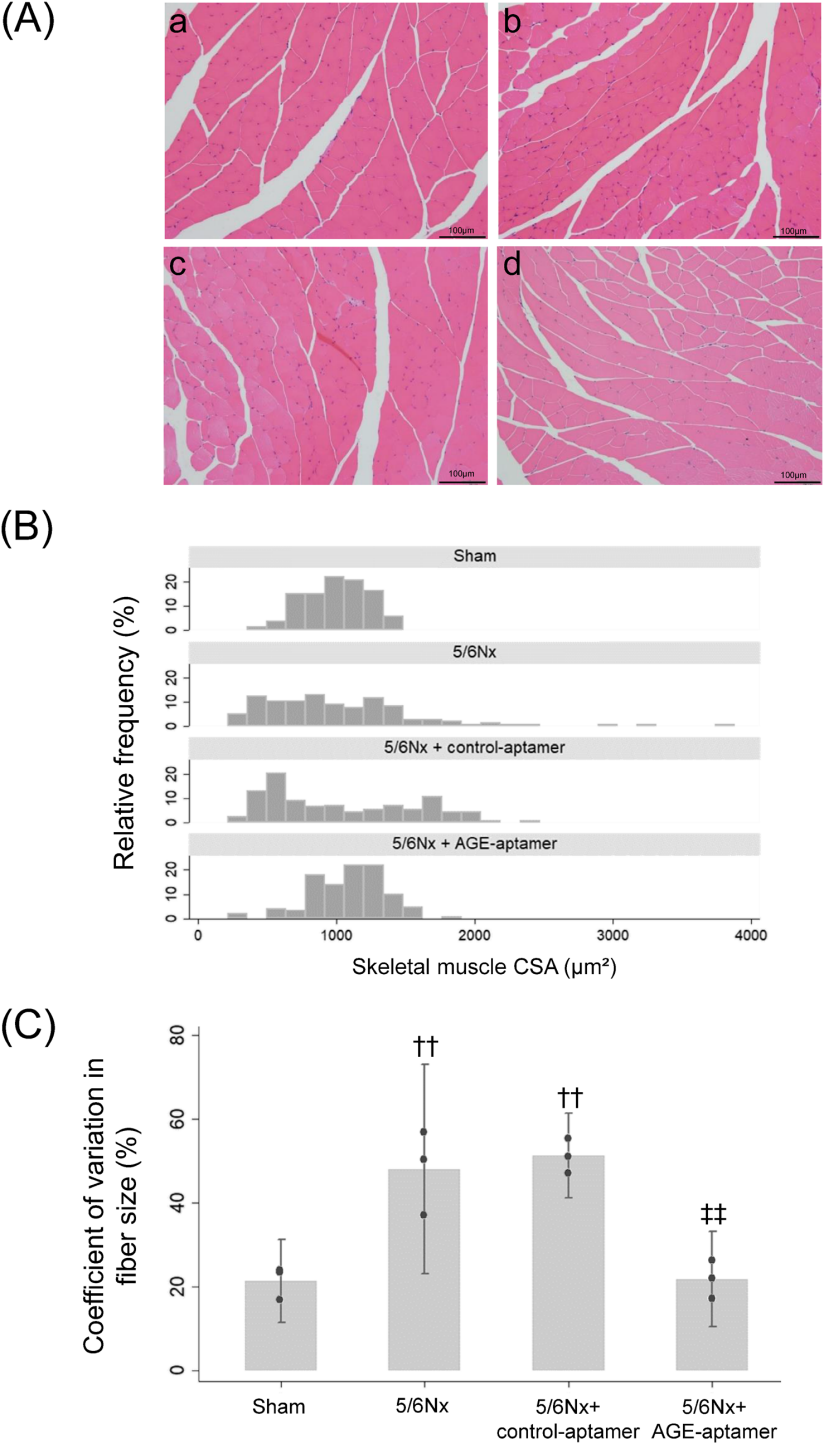
Morphological analysis of gastrocnemius muscles. (**A**) HE staining of the gastrocnemius muscles. × 20 magnification. a, Sham mice; b, 5/6Nx mice; c, 5/6Nx mice treated with control-aptamer; d, 5/6Nx mice treated with AGE-aptamer. (**B**) Histograms representing the size distribution of skeletal muscle fibers. (**C**) Coefficient of variation in fiber size. n = 3 per each group. ^††^p < 0.01 compared with sham mice. ^‡‡^p < 0.01 compared with 5/6Nx mice plus control-aptamer.

Histological analysis revealed that although most muscle fibers showed normal morphology with regular and polygonal muscle fibers in sham mice, muscle fibers in 5/6Nx mice showed irregular contours with random variation in fiber size, irrespective of the presence or absence of control-aptamer treatment (Fig. 3A,B). The coefficient of variations of cross-sectional area (CSA) were significantly higher in 5/6Nx and 5/6Nx-control aptamer mice than sham mice (p < 0.01), which were normalized by AGE-aptamer treatment (Fig. 3C).

### Effects of AGE-aptamer on the expression of the factors related to muscle regeneration and muscle atrophy in CKD mice

When evaluating sarcopenia and frailty, we need to closely observe on muscle regeneration as well as muscle atrophy^26^. Therefore, we conducted immunohistochemical analysis for MyoD, Pax7, and desmin, which are myogenic regulatory factors. Although desmin fluorescence immunointensity had no significant differences among the three groups (sham, 5/6Nx mice, and control-aptamer-treated 5/6Nx mice), AGE-aptamer administration significantly increased the desmin fluorescence immunointensity in the gastrocnemius muscles of control-aptamer-treated 5/6Nx mice (see Supplementary Figure S1 online). Meanwhile, MyoD and Pax7 were similar among the four groups (see Supplementary Figure S1 online).

In the western blot analysis of the gastrocnemius muscle, muscle-atrophy-related proteins such as myostatin and atrogin-1 were not different between the four groups (see Supplementary Figure S2 online). Consequently, Akt phosphorylation (p-Akt)/Akt ratio did not change in 5/6Nx mice, regardless of whether aptamer was administered or not (see Supplementary Figure S2 online).

### Effects of AGE-aptamer on capillary rarefaction of the gastrocnemius muscles in CKD mice

**Figure 4:**
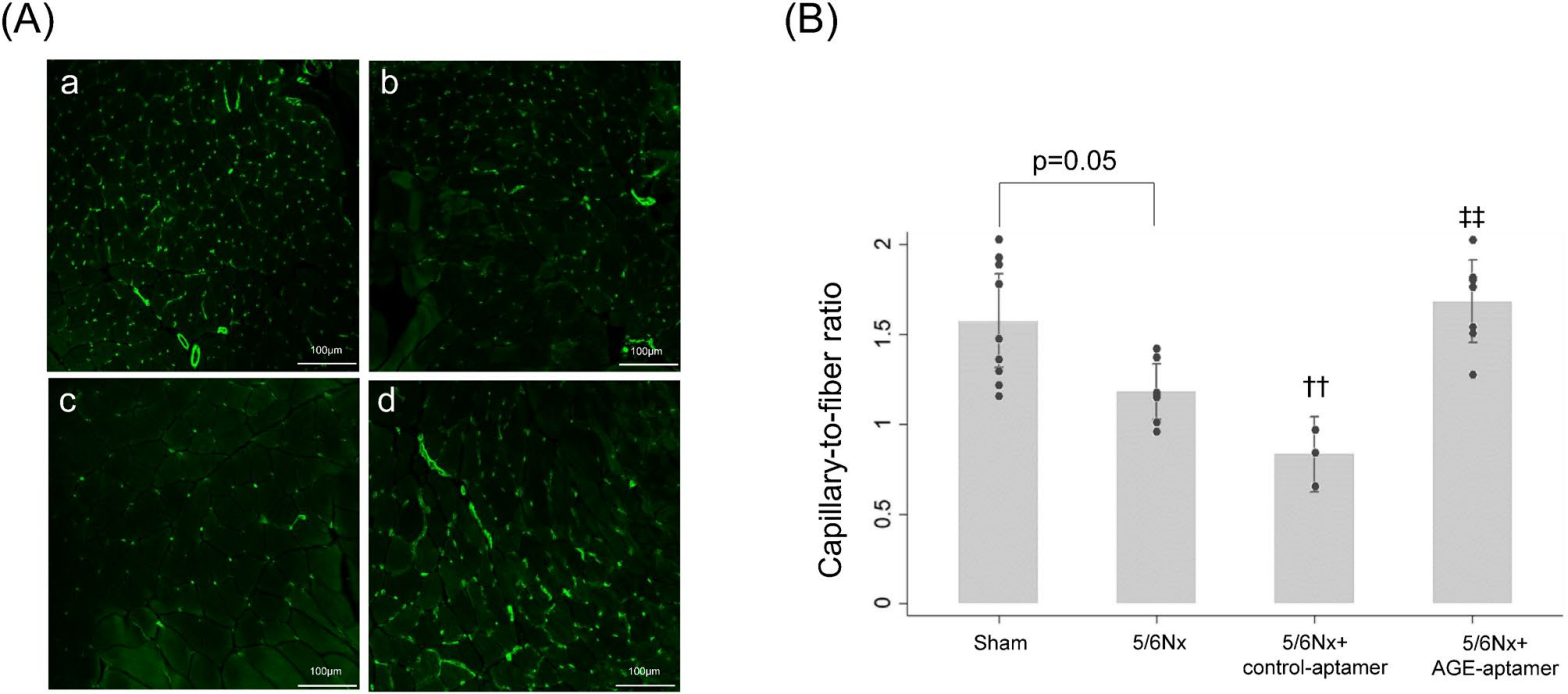
Capillary density in gastrocnemius muscles assessed by CD31 staining. (**A**) Representative microphotographs of CD31-positive capillaries. a, Sham mice; b, 5/6Nx mice; c, 5/6Nx mice treated with control-aptamer; d, 5/6Nx mice treated with AGE-aptamer. (**B**) Quantitative data of capillary density. n = 5–9 per each group. ^††^p < 0.01 compared with sham mice. ^‡‡^p < 0.01 compared with 5/6Nx mice plus control-aptamer.

As shown in Fig. 4A,B, capillary-to-fiber ratio in gastrocnemius muscles of 5/6Nx mice treated with control-aptamer was significantly lower than that of sham mice, which were restored by the treatment with AGE-aptamer (p < 0.01).

### Effects of AGE-aptamer on succinate dehydrogenase (SDH) immunostaining and PGC1-α levels in the gastrocnemius muscles of CKD mice

**Figure 5:**
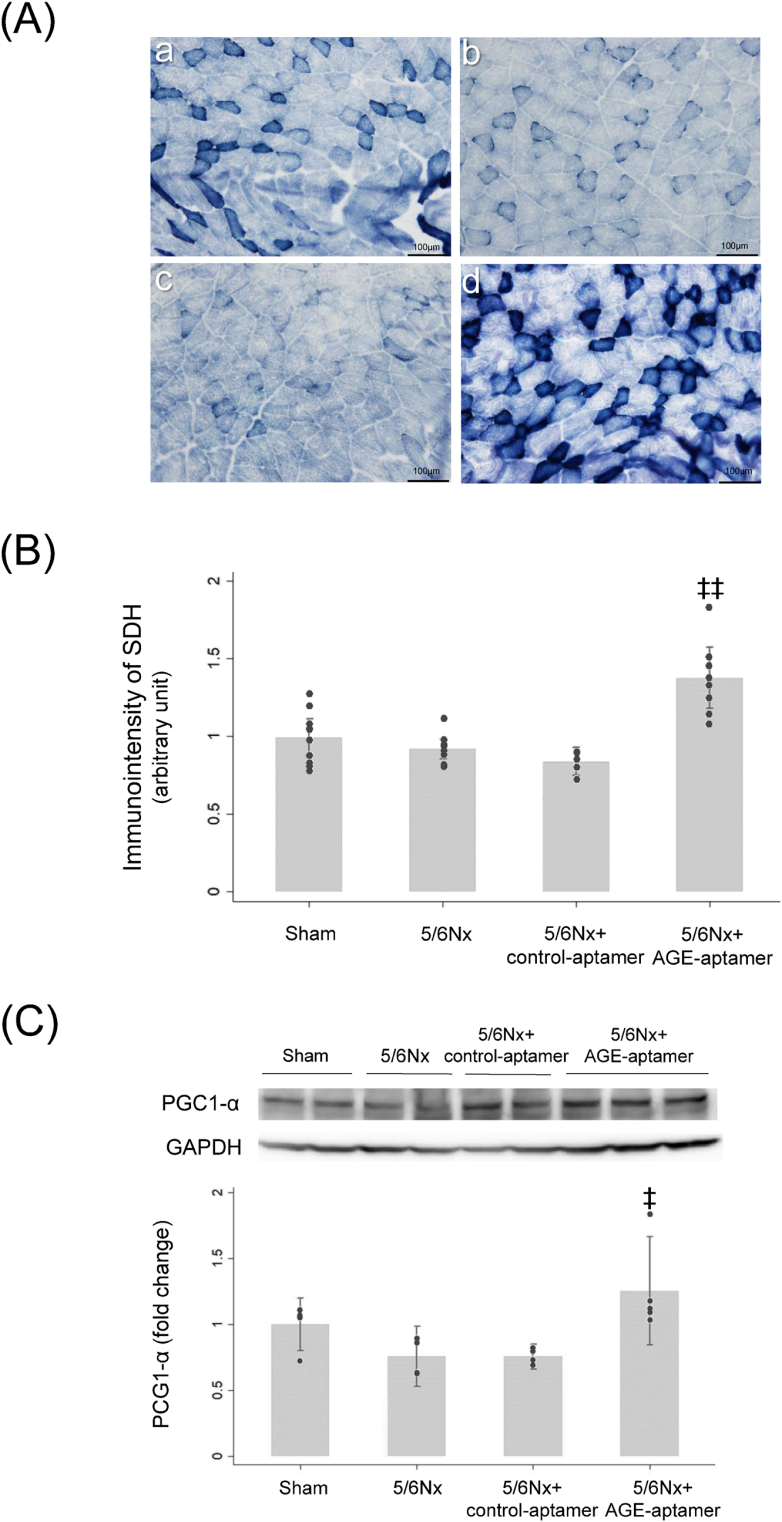
SDH immunostaining and PGC1-α expression levels in the gastrocnemius muscles. (**A**) Representative immunostaining of SDH. a, Sham mice; b, 5/6Nx mice; c, 5/6Nx mice treated with control-aptamer; d, 5/6Nx mice treated with AGE-aptamer. (**B**) Quantitative data of SDH immunostaining. n = 5–10 per each group. (**C**) Protein expression levels of PCG1-α. Upper panel shows representative bands of western blot analysis. Lower panel shows the quantitative data. n = 4–5 per each group. ‡ and ‡‡, p < 0.05 and p < 0.01 compared with 5/6Nx mice plus control-aptamer, respectively. Full-length blots are presented in Supplementary Figure S1.

As shown in Fig. 5A–C, although there were no significant differences in SDH immunostaining intensity and PGC1-α expression levels among the three groups, sham, 5/6Nx mice and 5/6Nx mice treated with control-aptamer, AGE-aptamer administration significantly increased the SDH immunostaining and PGC1-α expression levels in the gastrocnemius muscles of 5/6Nx mice treated with control-aptamer.

## Discussion

In the present study, we showed for the first time that (1) serum AGE levels were significantly increased according to the frailty status, and inversely associated with physical performance and physical activity in dialysis patients; (2) AGE were accumulated and RAGE expression were up-regulated in the gastrocnemius muscles of 5/6Nx mice in association with morphological abnormality, capillary rarefaction, and mitochondrial dysfunction; and (3) AGE-aptamer treatment ameliorated all of the deleterious effects on skeletal muscles. These observations suggest that AGE may trigger sarcopenia in CKD through capillary rarefaction and decreased mitochondrial biogenesis in the skeletal muscles, thereby being involved in frailty.

In the human study, we showed an association between AGE levels and frailty status. Moreover, among 5 components of frailty, slowness, weakness, weight loss, exhaustion, and decreased physical activity, we found here that high levels of AGE were significantly associated with slowness and weight loss. The present findings were consistent with the previous observations in older community-dwelling adults showing that elevated serum AGE levels were associated with slow walking speed^21^. It has been proposed that AGE can affect muscle function through various pathways in diabetic patients as well as older adults^27^. Accumulated AGE may contribute to increased connective tissue protein stiffness, thereby impairing muscle function^28^. In addition, AGE have been shown to induce skeletal muscle wasting via inflammatory reactions and/or endothelial dysfunction in the muscles through the interaction with RAGE^29^. Furthermore, Suliman et al*.* reported that circulating AGE levels were associated with malnutrition in patients with end-stage renal disease^30^. Since inflammation could contribute to malnutrition in patients with CKD via enhancement of protein catabolism and increased resting energy expenditure^31^. Taken together, our present study suggest the pathological role of AGE in frailty; AGE may play a role in slowness and weight loss by inducing inflammation and malnutrition. Circulating AGE levels were associated with low fat mass^32^. AGE levels have been shown to be correlated with inflammatory biomarkers, such as C-reactive protein and tumor necrosis-α in HD patients^12^. These observations support our speculation. In this study, we did not find the association of weakness with circulating levels of AGE, whose observations were contrast with the previous findings that AGE levels were associated with poor grip strength in older community-dwelling women^20^. The difference of subject population, such as sex, ethnicity, and background disease between our study and theirs may partly explain the discrepant results. Furthermore, although in this study, we found the inverse association of AGE with the average METs, AGE levels were not associated with the other two frailty components, exhaustion, and low physical activity. Serum levels of AGE were reported to be associated with weakness, exhaustion and low physical activity in older men, and odds of frailty increased with higher AGE values^23^. However, the association of AGE with frailty was lost after adjustments for comorbidities, including renal function. Therefore, slowness and weight loss may be more sensitive markers to detect the AGE-caused frailty in HD patients. As mentioned above, we also found an association between higher AGE levels and lower average METs, that is, subjective physical activity. One possible explanation for our finding is that high AGE levels could induce sarcopenia, which may limit the physical activity. In fact, resistance training has been reported to decrease the AGE levels in the tendons of older people^33^ and reduce the plasma AGE level in patients with type 2 diabetes^34^. In addition, increasing the skeletal muscle mass reduce renal damage via NO-dependent pathway^35^. Given that AGE levels were correlated inversely with average METs, exercise may reduce the AGE levels, which could contribute to the amelioration of endothelial dysfunction in patients with CKD. However, further studies are needed to elucidate this assumption.

Formation and accumulation of AGE have been progressed in diabetic patients, especially those with chronic kidney disease^9^. Since in this study, glycemic control in each group was relatively good and that there was no significant difference of glycemic control levels between prefrail and frail group , impact of diabetes on frailty may be unclear. However, AGE could reflect cumulative diabetic exposure, but not current glycemic control. Moreover CKD patients with diabetes mellitus are generally considered to exhibit higher AGE levels^25^ and more risk of frailty^36^, thus suggesting that AGE under either condition of diabetes or uremia may be involved in frailty. Further studies are needed to determine the impact of diabetes itself on frailty.

Johansen et al*.* reported that frailty in patients undergoing hemodialysis is prevalent by 68%^37^, which is higher than that obtained in our study (22%). The difference observed in the prevalence may be explained by the fact we included outpatients who could go to the hospital by themselves. Our human study results suggest that AGE might be an important factor in sarcopenia and frailty development in patients with CKD; however, we cannot draw any definite conclusion because of small sample size and selection bias in this study. Although further large-scaled studies are required to clarify this issue, an epidemiological study including 141 frail and 550 non frail subjects showing the association between RAGE and mortality in frail older patients^38^ could support our hypothesis.

Therefore, in order to further clarify the pathological role of AGE in skeletal muscle injury, we examined the effects of AGE-aptamer on gastrocnemius muscles in 5/6Nx mice, an animal model of CKD. To our knowledge, this is the first study to show that accumulation of AGE was enhanced in the gastrocnemius muscles of CKD model mice, which was prevented by AGE-aptamer. Since AGE were accumulated in skeletal muscles of aging rats, older people, and patients with fibromyalgia patients^39–41^, our present findings suggest that AGE accumulation in skeletal muscles is augmented by CKD that could accelerate premature aging^6^.

In the present study, we found that AGE-aptamer treatment significantly inhibited the increase in AGE accumulation and RAGE overexpression in the gastrocnemius muscles of 5/6Nx mice treated with control-aptamer and resultantly reduced the expression of desmin and the coefficient variation of CSA of skeletal muscles. Consistent with the results from previous reports, RAGE expression levels were up-regulated in conditions exposed to high AGE levels^42^. Furthermore, the expression of myogenin, which is a myoblast marker as with desmin, was decreased in the skeletal muscle cells of CKD mice^43^. Moreover, as mentioned in “Introduction”, AGE induced myogenic dysfunction, resulting in skeletal muscle atrophy and dysfunction in diabetic mice via a RAGE-mediated signaling pathway^24^. Therefore, the AGE-RAGE axis has an important role in the development of sarcopenia and frailty via an impaired myogenic response. Since atrophied muscle fibers with a random distribution of fiber size and shape within the skeletal muscles were characteristic features of uremic myopathy and/or sarcopenia^44^, AGE could cause morphological alterations in the gastrocnemius muscles in CKD mice, which may partly explain the link between AGE levels and slowness in our HD patients.

We found here that AGE-aptamer restored capillary rarefaction in the gastrocnemius muscles in 5/6Nx mice. We, along with others, have previously shown that AGE inhibit nitric oxide (NO) synthesis and bioavailability, thereby being involved in endothelial injury and microvascular rarefaction in animal models of CKD^45,46^. We previously reported that AGE levels were positively correlated with endogenous NO synthase inhibitor, asymmetric dimethylarginine (ADMA), suggesting that AGE could contribute to endothelial dysfunction in patients undergoing hemodialysis^47^. In addition, accumulated ADMA could be involved in rarefaction or dysfunction of renal capillaries in CKD animal models^48^. Therefore, the AGE-ADMA axis may also contribute to capillary rarefaction and ischemia in the gastrocnemius muscles of CKD mice via decreased NO synthesis and bioavailability. Low capillary-to-fiber ratio was observed in biopsy samples of vastus lateralis muscle of HD patients^49^, further supporting the pathological role of capillary rarefaction in AGE-associated skeletal muscle injury.

Impaired mitochondrial respiratory function, reduced muscle mitochondrial mass, and decreased energy production in the skeletal muscles play important roles in uremic sarcopenia^50^. We found here that AGE-aptamer significantly ameliorated the decrease in SDH activity and PGC1-α, a marker of mitochondrial biosynthesis in the gastrocnemius muscles of 5/6Nx mice treated with control-aptamer. Several studies have shown that mitochondrial dysfunction in the skeletal muscles were associated with sarcopenia in both patients^50,51^ and animal models^52–54^ with CKD. AGE may impair mitochondrial energy production and biosynthesis partly by reducing the SDH activity and PGC1-α levels^53,54^.

In addition to the limitation of small sample size and selection bias in this clinical study, this study has several limitations. First, our CKD mice did not display skeletal muscle atrophy, and expression levels of muscle atrophy-related proteins were not altered. Skeletal muscle atrophy has been shown to be observed in 5/6Nx mice 1 year after the nephrectomy^52^. Therefore, it would be interesting to examine the long-term effects of AGE-aptamer on skeletal muscle atrophy in our CKD model. Second, although AGE-aptamer reduced the AGE accumulation in gastrocnemius muscles in 5/6Nx mice treated with control-aptamer, it did not affect renal function in these mice. The findings suggest that AGE accumulated in the skeletal muscles rather than renal dysfunction itself may play a pathological role in uremic myopathy and/or sarcopenia in CKD. However, AGE-aptamer administration reportedly decreases the plasma level of BUN and creatinine in diabetic mice^55^. Therefore, it is possible that the present study may overestimate the renal function of the control-aptamer-treated 5/6Nx mice because their survival rates were lower than those in AGE-aptamer-treated 5/6Nx mice (69.2% [9 of 13] vs. 93.3% [14 of 15]). In our study, mice with poor renal function possibly may die before the evaluation of aptamer administration that could explain the discrepant results between previous report and the present study.

In conclusion, our present study suggests that circulating AGE levels are increased according to the frailty status and may be involved in altered morphology, capillary rarefaction, and mitochondrial dysfunction in the skeletal muscles of CKD animals. AGE in the skeletal muscles may be a therapeutic target for sarcopenia and frailty in CKD.

## Materials and methods

### Subjects

Thirty-seven patients who underwent maintenance hemodialysis (HD) at the dialysis center of our hospital were enrolled in the present study. We excluded any hemodialysis patients with malignancy, active inflammation, steroid therapy, or primary skeletal muscle disease. Informed consent was obtained from all the participants. This study was approved by the Ethics Committee of Juntendo University and conducted in accordance with the principles of the Declaration of Helsinki.

### Measurement of clinical and biochemical parameters

Blood samples were obtained from the arterial hemodialysis line before the start of hemodialysis session, and serum was immediately frozen at − 80 °C until analyzed. Serum levels of AGE were determined by enzyme-linked immunosorbent assay as described previously^56^. In this study, one unit (U) corresponds to half maximal (50%) inhibitory concentration of AGE. Blood biochemistry was measured using standard laboratory methods^57^, and blood pressure was evaluated as reported previously^57^. The Geriatric Nutritional Risk Index (GNRI) was used to assess nutrition status^58^. GNRI is calculated from serum albumin level and body weight using the following formula: GNRI = [1.489 × albumin (g/dl)] + [41.7 × (body weight/ideal body weight)]. The ideal body weight of each patient was calculated based on their height, with a target body mass index (BMI; weight in kg divided by the square of the height in meters) of 22^58^.

### Evaluation of frailty

Physical frailty was characterized by the following five conditions: slowness, weakness, weight loss, exhaustion, and low physical activity according to the original study of Fried et al*.* with a slight modification^59,60^. Participants were classified as frail if they met three or more of these conditions, and as prefrail if they met one or two conditions, and as control if no condition was present. Slowness was defined based on walking speed, with a cutoff level < 1.0 m/s. Weakness was measured by grip strength, and cutoff level was < 26 kg for men and < 18 kg for women. Weight loss was assessed by a response of “yes” to the question, “Have you lost 2 kg or more in the past six months?” Exhaustion was considered to be present if the participant responded with “yes” to the following question: “In the last two weeks, have you felt tired for no reason?” Physical activity was evaluated by asking the following questions about time spent engaged in sports and exercise: (1) “Do you engage in moderate levels of physical exercise or sports aimed at health?” and (2) “Do you engage in low levels of physical exercise aimed at health?” Participants who answered “no” to both of these questions were classified as low activity.

### Measurement of physical performance and activity

Physical performance was evaluated based on grip strength, one-leg standing balance, and TUG test. Grip strength was measured using a handheld dynamometer with participants in a standing position. For the analysis, we used higher value (kg) of two trials with a 30-s rest period in between. One-leg standing balance test required the participants to stand unassisted on any one leg as long as possible. The participants carried out two timed trials, and the best time was used for the one-leg standing balance. The TUG test required the participants to stand up, walk 3 m, turn, and return to the start position.

To measure activity strength and exercise quantity, we used a 3-axis wristband-type acceleration sensor (wristband-type life recorder UW-301, A&D Company Limited, Japan). The participants wore the activity meter for 7 consecutive days. The average daily step count and METs per day were measured.

### Preparation of DNA-aptamer directed against AGE (AGE-aptamer)

AGE-aptamer and control-aptamer were prepared as previously described^55^. In brief, AGE-aptamers were selected from pools of synthetic DNA templates (106-mer) with 56 random nucleotides by systematic evolution of ligands by exponential enrichment (SELEX)^61^.

### Animal experiments

Male C57BL/6 mice were purchased from CLEA Japan (Tokyo, Japan) and maintained in a specific pathogen-free mouse center at Juntendo University. CKD was induced by 5/6 nephrectomy (Nx) under anesthesia consisting of 0.3 mg/kg medetomidine, 4 mg/kg midazolam, and 5 mg/kg butorphanol by intraperitoneal injection. In one group, right kidney was removed, and upper and lower thirds of left kidney were resected. The other group consisted of mice that underwent sham procedures, including decapsulation of both kidneys. One week after the 5/6Nx, the mice were divided into three groups: mice treated with continuous intraperitoneal infusion (0.136 μg/day) of either AGE-aptamer or control-aptamer by an osmotic pump (model 2006; ALZET, Cupertino, CA, USA) or without treatment. All the mice were killed 7 weeks after the surgery, and blood and gastrocnemius muscles were obtained for morphological, immunohistochemical, and biochemical analyses. Body weights of mice were recorded just before the tissue removal. Plasma levels of blood urea nitrogen (BUN) and creatinine were measured using a Fujidrychem7000 and DRI-CHEM slide system (FUJIFILM, Kanagawa, Japan) following the manufacturer’s protocol. All animal experiments and procedures were approved by Juntendo University, and conducted accordance with the guidelines of Juntendo University for the care and use of laboratory animals.

### Morphological analysis

At the end of the experiments, gastrocnemius muscles were obtained from mice under anesthesia. Tissues were then fixed with 4% paraformaldehyde, embedded in paraffin, and sectioned at 10 μm intervals and mounted on glass slides. The sections were stained with hematoxylin and eosin (HE) for morphological analysis. CSA of at least 50 muscle fibers from each mouse was determined using ImageJ software (U.S. National Institutes of Health, USA).

### Immunohistochemical analysis

The sections were incubated with monoclonal antibody raised against AGE^62^ after pretreatment with blocking agents, and the reactions were visualized with a Mouse on Mouse Basic Kit (BMK-2202; Vector Laboratories, Burlingame, CA)^63^.

Frozen tissues were used for RAGE, MyoD, Pax7, desmin, CD31 and SDH staining. The frozen sections were incubated with blocking reagents (Dako, Glostrup, Denmark) for 60 min and then with rabbit polyclonal antibody raised against mouse RAGE (1:200 dilution; Abcam, ab3611), MyoD (1:250 dilution; Abcam, ab64159), Pax7 (1:20 dilution; Abcam, ab187339), desmin (1:200 dilution; Thermo Fisher, PA5-16075), and CD31 (1:50 dilution; Abcam, ab28364) overnight at 4 °C. The sections were then incubated with Alexa-Fluor 488 anti-rabbit antibody (1:300 dilution; Abcam) for 60 min at room temperature, and visualized as described previously^64^. Fluorescence intensity was quantified by converting green fluorescence signal from raw images, obtained under identical exposure conditions, to grayscale and analyzing the pixel intensity by KS400 imaging system (Car Zeiss Meditec, Obercocken, Germany). Capillary-to-fiber ratio was defined as total number of capillaries divided by total number of fibers within the sample.

To evaluate the SDH activity in gastrocnemius muscles, the frozen sections were incubated at 37 °C for 45 min in a medium containing nitroblue tetrazolium (1.2 M), sodium succinate (0.2 M), and sodium phosphate buffer pH 7.5 (0.2 M). Immunohistoreactivity in approximately 500 fibers from 3–5 different fields in each sample was measured by KS400 imaging system.

### Western blot analysis

Proteins were extracted from the gastrocnemius muscles using RIPA buffer with protease and phosphatase inhibitors, and subjected for western blot analysis using polyclonal antibody raised against myostatin (Abcam, ab71808), Fbx32/atrogin-1 (Abcam, ab168372), Akt (Cell Signaling Technology, #4691), p-Akt (Cell Signaling Technology, #9271), peroxisome proliferator-activated receptor γ coactivator-1 (PGC1-α) (Abcam, ab55481) and GAPDH (Abcam, ab9484) as described previously^65^.

### Statistical analysis

Data were shown as mean ± standard deviation. Differences were analyzed using the following statistical tests. Continuous variables were compared using analysis of variance, and categorical variables were analyzed with the χ^2^ test of Fisher’s exact test. One-way analysis of variance (ANOVA) with a Bonferroni posttest was used to test for differences among groups when appropriate. P-values < 0.05 were considered statistically significant. All analyses were carried out using Stata SE, version 12.0 and version 14.2 (Stata Corp LP, College Station, TX, USA).

## Supplementary information

Supplementary Information.
