# Supplementary material for: Association of advanced glycation end products with sarcopenia and frailty in chronic kidney disease
Source: Sci Rep. 2020 Oct 19;10:17647. doi: 10.1038/s41598-020-74673-x (PMC7573579; doi:10.1038/s41598-020-74673-x)
Supplement: Supplementary file 1 — Supplementary Information. [file 41598_2020_74673_MOESM1_ESM.pdf]

Association of advanced glycation end products with sarcopenia and frailty in chronic kidney disease

Junko Yabuuchi[1], Seiji Ueda[1*], Sho-ichi Yamagishi[2], Nao Nohara[1], Hajime Nagasawa[1], Keiichi Wakabayashi[1], Takanori Matsui[3], Higashimoto Yuichiro[4], Tomoyasu Kadoguchi[5], Tomoyuki Otsuka[1], Tomohito Gohda[1], and Yusuke Suzuki[1]

Supplementary Table S1. Association of serum AGE levels with frailty components

Criteria	Number	AGE	p-value
Slowness			0.02
(+)	5	1.36±1.72	
(-)	25	0.56±0.24	
Weakness			0.27
(+)	15	0.83±1.02	
(-)	14	0.51±0.23	
Weight loss			<0.01
(+)	6	1.41±1.51	
(-)	27	0.51±0.21	
Exhaustion			0.43
(+)	6	0.79±0.31	
(-)	27	0.65±0.79	
Low physical activity			0.34
(+)	27	0.72±0.79	
(-)	8	0.45±0.14	

Data are shown as mean ± standard deviation.

Supplementary Figure S1.

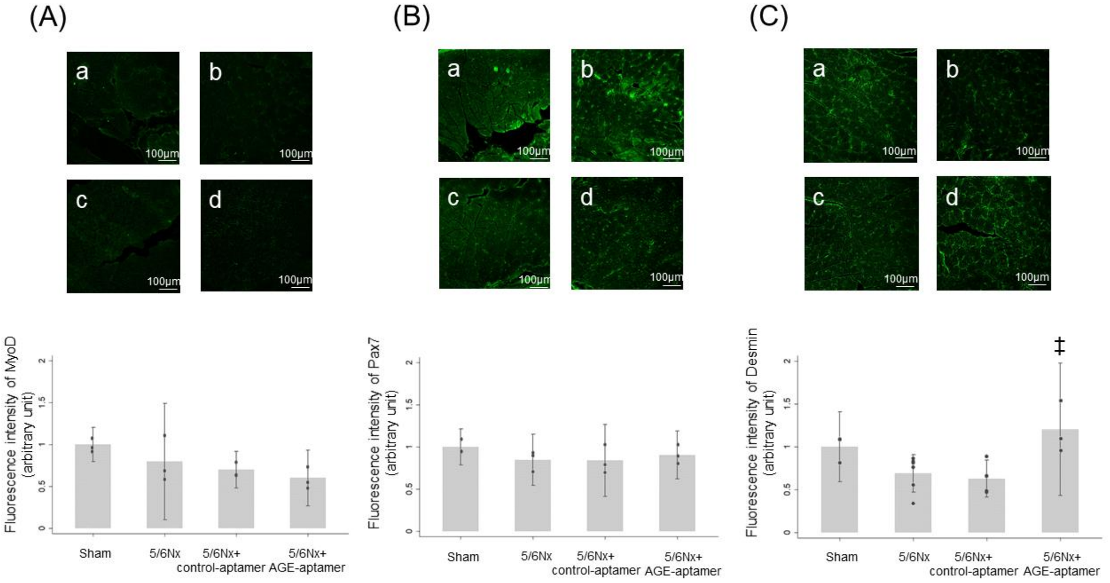

Supplementary Figure S2.

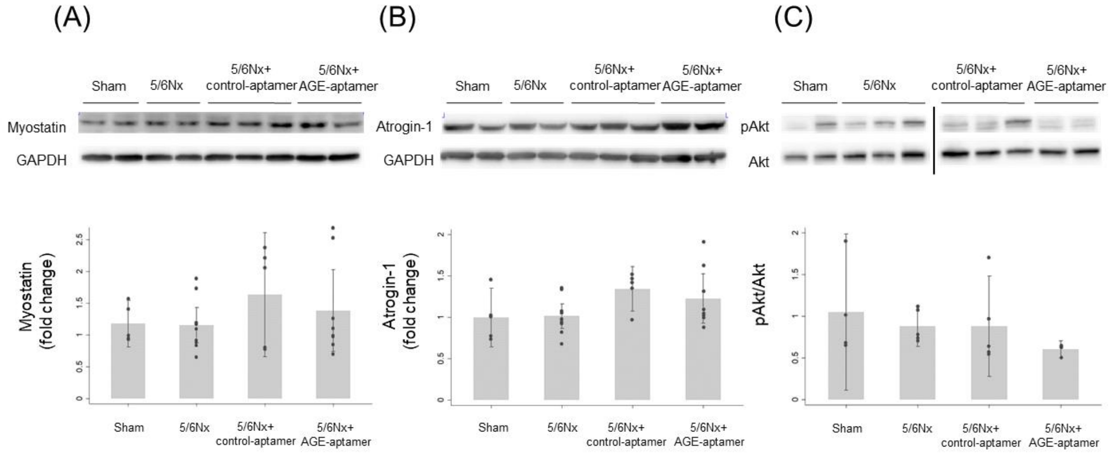

Supplementary Figure S3.

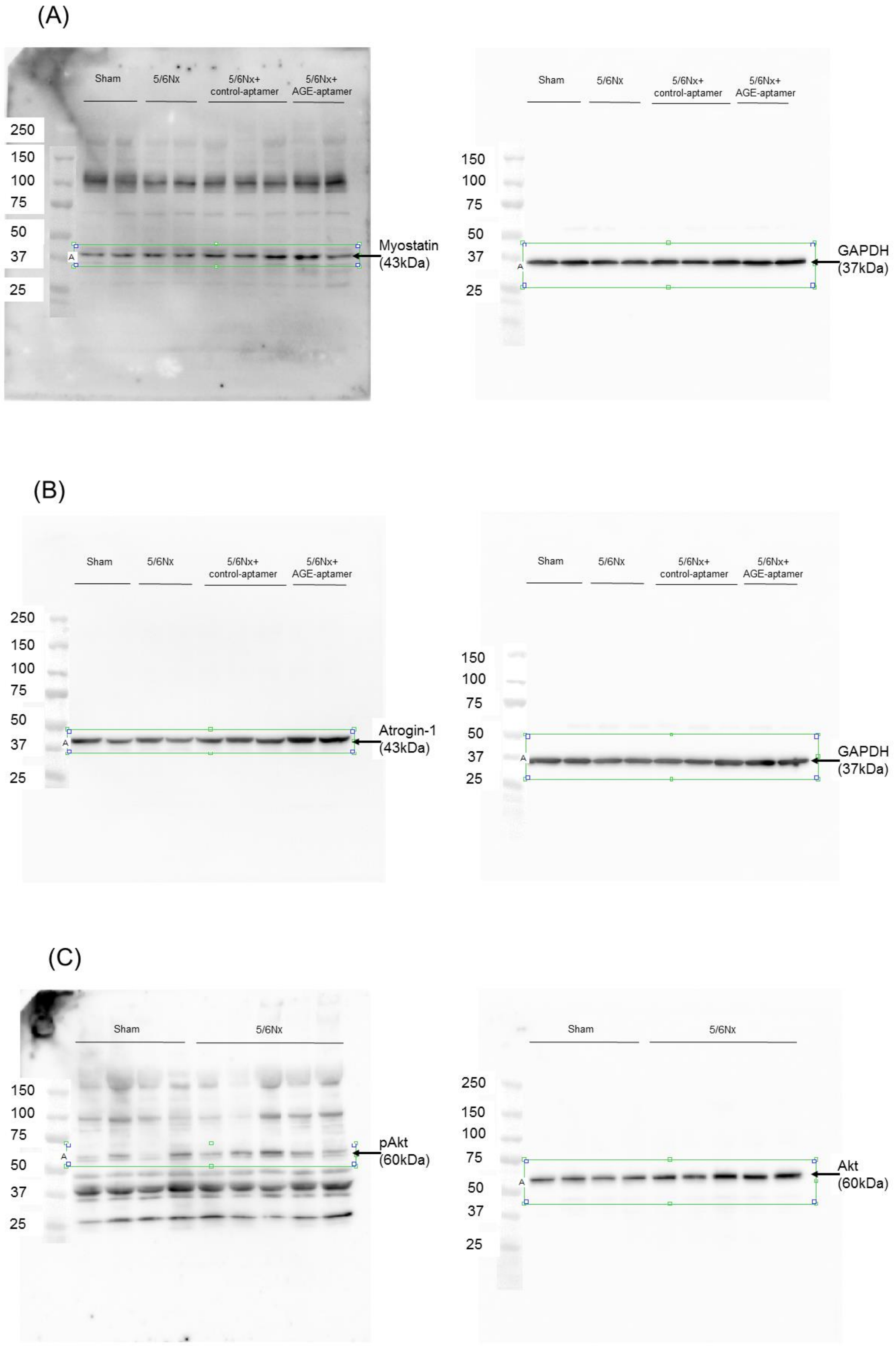

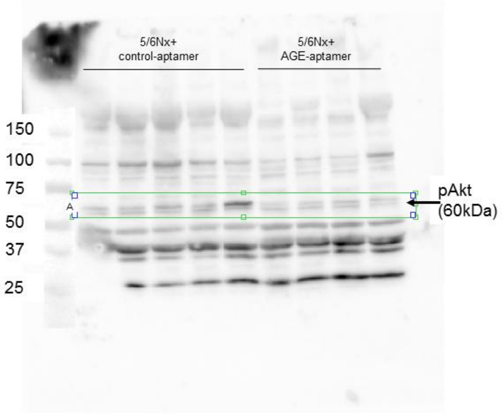
5/6Nx+
control-aptamer
5/6Nx+
AGE-aptamer
150
100
75
50
37
25
pAkt
(60kDa)

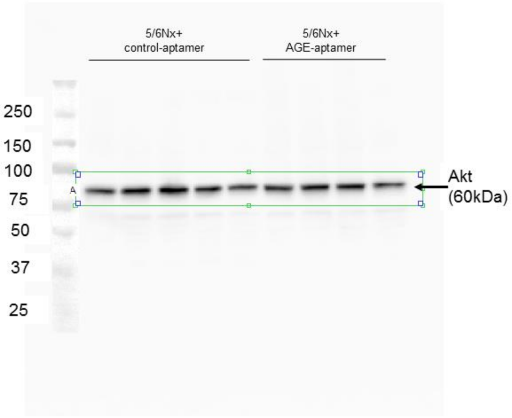
5/6Nx+
control-aptamer
5/6Nx+
AGE-aptamer
250
150
100
75
50
37
25
Akt
(60kDa)

Supplementary Figure S4.

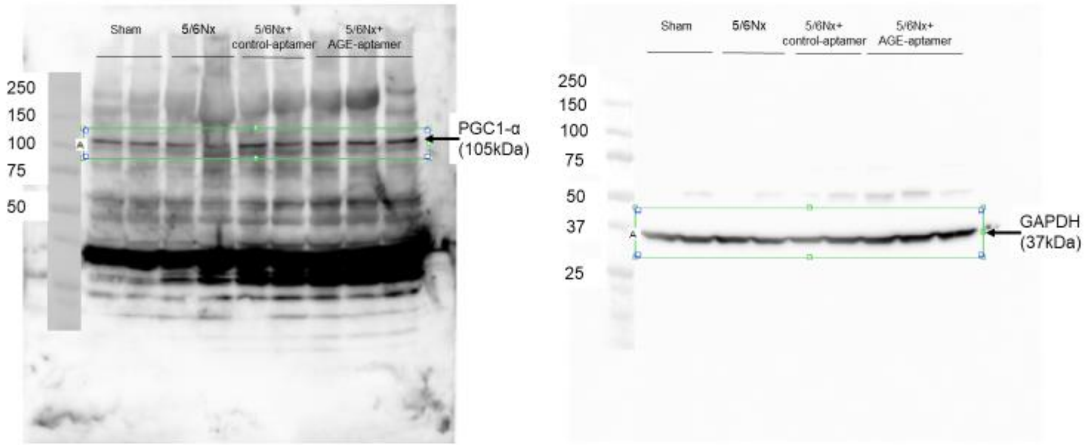

Supplementary figure legends

Supplementary Figure S1. Factors related to muscle regeneration in the gastrocnemius muscles.

(A) Representative microphotographs of MyoD immunostaining of the gastrocnemius muscle and quantitative data of MyoD staining. a, Sham mice; b, 5/6Nx mice; c, 5/6Nx mice treated with control-aptamer; d, 5/6Nx mice treated with AGE-aptamer. n=3 per each group. (B) Representative microphotographs of Pax7 immunostaining of the gastrocnemius muscle and quantitative data of Pax7 staining. a, Sham mice; b, 5/6Nx mice; c, 5/6Nx mice treated with control-aptamer; d, 5/6Nx mice treated with AGE-aptamer. n=3 per each group. (C) Representative microphotographs of desmin immunostaining of the gastrocnemius muscle and quantitative data of desmin staining. a, Sham mice; b, 5/6Nx mice; c, 5/6Nx mice treated with control-aptamer; d, 5/6Nx mice treated with AGE-aptamer. n=3-6 per each group. ‡p <0.05 compared with 5/6Nx mice plus control-aptamer.

Supplementary Figure S2. Factors related to muscle atrophy in the gastrocnemius muscles.

(A) myostatin; (B) atrogin-1; (C) phosphorylated Akt. Upper panel shows the representative bands of western blot analysis. Cropped gels are shown in panel (C) and the gels were run under the same conditions. Lower panel shows the quantitative data. n=4-9 per each group. Full-length blots are presented in Supplementary Figure S3.

Supplementary Figure S3. Full scans of panel from Supplementary Figure S2.

(A) myostatin; (B) atrogin-1; (C) phosphorylated Akt. The intensity of each band was detected and quantified using Vilber-Lourmat FUSION (M&S Instruments Inc., Osaka, Japan).

Supplementary Figure S4. Full scans of the panel from Figure 5C.

The intensity of each band was detected and quantified using Vilber-Lourmat FUSION (M&S Instruments Inc., Osaka, Japan).
